# Ethyl Pyruvate Decreases Collagen Synthesis and Upregulates MMP Activity in Keloid Fibroblasts and Keloid Spheroids

**DOI:** 10.3390/ijms25115844

**Published:** 2024-05-28

**Authors:** Wooyeol Baek, Seonghyuk Park, Youngdae Lee, Hyun Roh, Chae-Ok Yun, Tai Suk Roh, Won Jai Lee

**Affiliations:** 1 Department of Plastic & Reconstructive Surgery, Severance Hospital, Yonsei University College of Medicine, Seoul 03722, Republic of Korea; 2 Institute for Human Tissue Restoration, Yonsei University College of Medicine, Seoul 03722, Republic of Korea; 3 Department of Bioengineering, College of Engineering, Hanyang University, Seoul 04763, Republic of Korea; 4 Institute of Nano Science and Technology (INST), Hanyang University, Seoul 04763, Republic of Korea

**Keywords:** high-mobility group box 1 (HMGB1), ethyl pyruvate, keloid

## Abstract

Keloids, marked by abnormal cellular proliferation and excessive extracellular matrix (ECM) accumulation, pose significant therapeutic challenges. Ethyl pyruvate (EP), an inhibitor of the high-mobility group box 1 (HMGB1) and TGF-β1 pathways, has emerged as a potential anti-fibrotic agent. Our research evaluated EP’s effects on keloid fibroblast (KF) proliferation and ECM production, employing both in vitro cell cultures and ex vivo patient-derived keloid spheroids. We also analyzed the expression levels of ECM components in keloid tissue spheroids treated with EP through immunohistochemistry. Findings revealed that EP treatment impedes the nuclear translocation of HMGB1 and diminishes KF proliferation. Additionally, EP significantly lowered mRNA and protein levels of collagen I and III by attenuating TGF-β1 and pSmad2/3 complex expression in both human dermal fibroblasts and KFs. Moreover, metalloproteinase I (MMP-1) and MMP-3 mRNA levels saw a notable increase following EP administration. In keloid spheroids, EP induced a dose-dependent reduction in ECM component expression. Immunohistochemical and western blot analyses confirmed significant declines in collagen I, collagen III, fibronectin, elastin, TGF-β, AKT, and ERK 1/2 expression levels. These outcomes underscore EP’s antifibrotic potential, suggesting its viability as a therapeutic approach for keloids.

## 1. Introduction

Keloids, considered benign fibroproliferative tumors, represent a form of pathological scarring of the skin characterized by an excessive accumulation of extracellular matrix (ECM) components such as collagen, fibronectin, and elastin [1]. Notably, keloids extend beyond the original boundary of injury and rarely regress spontaneously [2,3]. They can significantly affect the quality of life due to aesthetic concerns caused by bodily disfigurement and often accompany symptoms like pain and pruritus. Despite the great interest in managing keloids, no definitive therapeutic options are currently available, partly because the exact pathogenesis of the condition remains unclear. The formation of keloids is influenced by a multifactorial etiology, including genetic and environmental factors, inflammation, mechanical stress, and even the microbiome.

One of the most widely recognized mechanisms in the pathophysiology of keloids is the excessive accumulation of ECM, caused by an increased number of keloid fibroblasts (KFs) [2,3,4,5,6,7]. It is thought that both an increase in cellular proliferation and decreased apoptosis contribute to the overpopulation of KFs. Over-proliferation is believed to be driven by various growth factors, including transforming growth factor-beta 1 (TGF-β1), platelet-derived growth factor (PDGF), and endothelial growth factor (EGF) [8,9]. Notably, TGF-β1 increases the synthesis of collagen and the proliferation of KFs via the regulation of ECM-degrading enzymes, including matrix metalloproteinases (MMPs) and tissue inhibitor of metalloproteinases 1 (TIMP1) [10].

Ethyl pyruvate (EP) is a single fatty acid lipid derived from pyruvic acid. Due to its low cost and low toxicity, EP has been considered for potential use in a wide range of areas within the biomedical field. Previous research has highlighted the anti-apoptotic, anti-oxidative, anti-inflammatory, and anti-fibrotic properties of this molecule [11]. Its effects on suppressing fibrotic changes have been observed in various organs. EP has been shown to reduce fibrotic changes in myocardial tissue in response to treatment with doxorubicin, namely by reducing the expression of TGF-β1, collagen I/III, MMP-2/9, and TIMP-1/2 [12]. Furthermore, the injection of EP into an in-vivo liver fibrosis rat model induced by CCl4 resulted in reduced fibrosis and recovery of liver function [13]. For pulmonary fibrosis, Hamada et al. discovered that EP treatment also had protective effects against bleomycin-induced lung injury in a rat model, possibly by inhibiting the level of high-mobility group box 1 (HMGB1) [14].

Recently, dermal inflammation caused by various local factors has also been introduced as a new aspect of keloid pathophysiology [15]. Treatment with lipopolysaccharide (LPS) induced inflammation in fibroblasts, resulting in keloid formation through the activation of HMGB1 [16].

HMGB1 is a nuclear protein that primarily acts as a DNA chaperone in DNA replication, recombination, transcription, and repair [17]. After cell activation or damage, HMGB1 is released from the nucleus into the cytosol or extracellular space, functioning as a damage-associated molecular pattern (DAMP) protein [18]. Overexpression of cytosolic HMGB1 promotes autophagy and inflammation, which are associated with increased cell proliferation, mobility, angiogenesis, and resistance to apoptosis [19,20,21]. In our previous study, LPS-induced inflammation in fibroblasts increased the activation of HMGB1, subsequently increasing keloid formation through the Erk1/2, Akt, and NF-κB signaling pathways [16,22,23]. 

In this study, we aim to explore the therapeutic potential of EP for the treatment of pathological fibrosis, specifically keloid formation, and to identify its potential for further clinical application. EP has been identified to decrease the effects of HMGB1, which inhibits chemotaxis and mitotic activity in the extracellular space [24,25]. By inhibiting the cytoplasmic translocation of HMGB1, EP may protect against inflammation, pathological fibrosis, and oncogenesis, potentially disrupting keloid progression and attenuating fibrosis in keloids [26,27].

To examine this hypothesis, we investigated the effects of EP on the proliferation of KFs and the production of the ECM following the activation of inflammatory and profibrotic pathways by treatments with LPS and TGF-β1 in human dermal fibroblasts (HDF). Additionally, we assessed the proliferation and ECM production in both KFs and patient-derived keloid spheroids. Furthermore, expression levels of type-I and -III collagen, fibronectin, and elastin were analyzed in keloid tissue spheroids treated with EP through immunohistochemistry, providing a comprehensive insight into the compound’s capacity to attenuate fibrosis and potentially offer a novel therapeutic avenue for keloid management.

## 2. Results

### 2.1. Ethyl Pyruvate Reduces Translocation of Nuclear HMGB1

**Figure 1:**
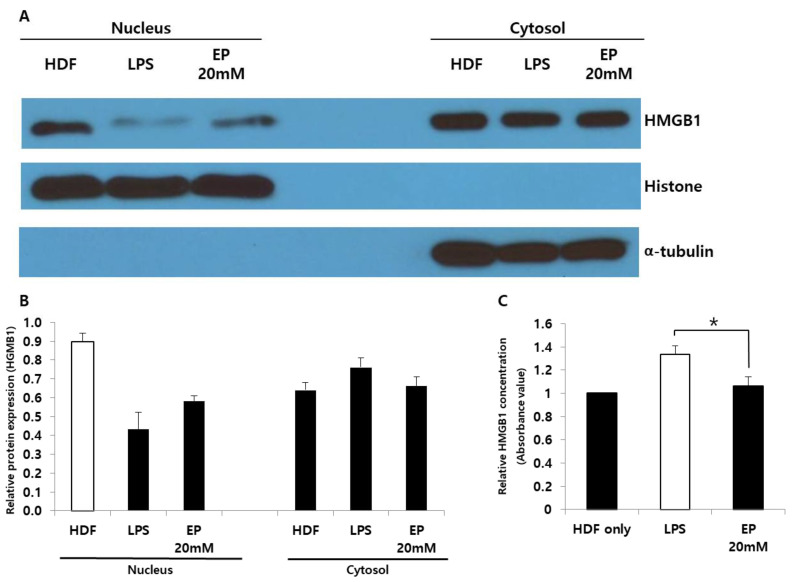
Effects of ethyl pyruvate on HMGB1 expression in LPS-treated HDFs. (**A**) Western blotting confirmation of the nucleus and cytosolic HMGB1 expression in LPS-treated HDFs. α-tubulin was used as endogenous control for cytosolic protein; histone was used as endogenous control for nuclei protein. (**B**) Quantitative analysis of western blotting. Although not statistically significant, nuclear protein expression of HMGB1 was increased by treatment of ethyl pyruvate in LPS-treated HDFs. In contrast, cytosolic protein expression of HMGB1 was relatively decreased by ethyl pyruvate treatment. (**C**) ELISA analysis of extracellular HMGB1 concentration. EP treatment significantly reduced extracellular HMGB1 compared to LPS-treated HDFs (* *p* < 0.05).

In our previous study, we investigated the role of HMGB1, which, when relocalized to the cytosol or extracellular spaces, acts as a profibrotic molecule. We subjected HDFs to cell fractionation to isolate nuclear and cytoplasmic fractions and performed western blotting analysis. This was to determine if nuclear-cytoplasmic translocation occurred during stimulation by an inflammatory mediator, such as LPS (500 ng), and to assess whether this effect could be reversed by EP treatments (20 mM). As depicted in Figure 1A, HMGB1 protein expression levels in the nuclear fractions decreased after 48 h of LPS treatment, while cytoplasmic HMGB1 increased (Figure 1B). However, treatment with EP in LPS-treated HDFs increased nuclear HMGB1 protein expression and decreased cytoplasmic HMGB1, although the differences were not statistically significant. The difference in the extracellular amount of HMGB1 was confirmed by ELISA (Figure 1C). Data showed that extracellular expression of HMGB1 was significantly reduced in HDFs treated with both EP and LPS compared to those treated with LPS alone (* *p* < 0.05). Our results indicate that EP treatment of LPS-stimulated HDFs resulted in the relocalization of nuclear HMGB1 from the cytosol back to the nucleus and reduced the expression of extracellular HMGB1 protein.

### 2.2. Ethyl Pyruvate Reduces Proliferation of TGF-β1 Induced HDFs and KFs

**Figure 2:**
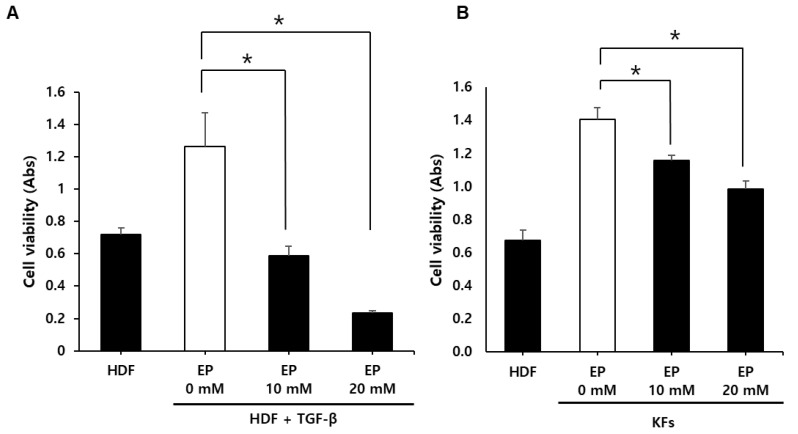
Effects of ethyl pyruvate on cellular viability of TGF-β1-treated HDFs (**A**) and KFs (**B**). MTT cell proliferation assay shows that ethyl pyruvate significantly reduced the proliferation of both TGF-β1-treated HDFs and KFs in a dose-dependent manner (* *p* < 0.05).

To assess the impact of EP on cellular proliferation, we incubated HDFs treated with 10 ng/mL TGF-β1 and KFs with various concentrations of EP for the proliferation assay. Subsequently, we conducted a 3-(4,5-dimethylthiazol-2-yl)-2,5-diphenyltetrazolium bromide (MTT) cell proliferation assay. In HDFs treated with TGF-β1 [28], a significant reduction in proliferation was observed following EP treatment, with the effects being dose-dependent (Figure 2A). Similarly, in KFs, proliferation significantly decreased after treatment with all examined concentrations of EP (Figure 2B, * *p* < 0.05). These results suggest that EP effectively reduces the cellular proliferation of both TGF-β1-treated HDFs and KFs.

### 2.3. Effect of Ethyl Pyruvate on mRNA Expression of Collagen I and Collagen III in HDFs and KFs

**Figure 3:**
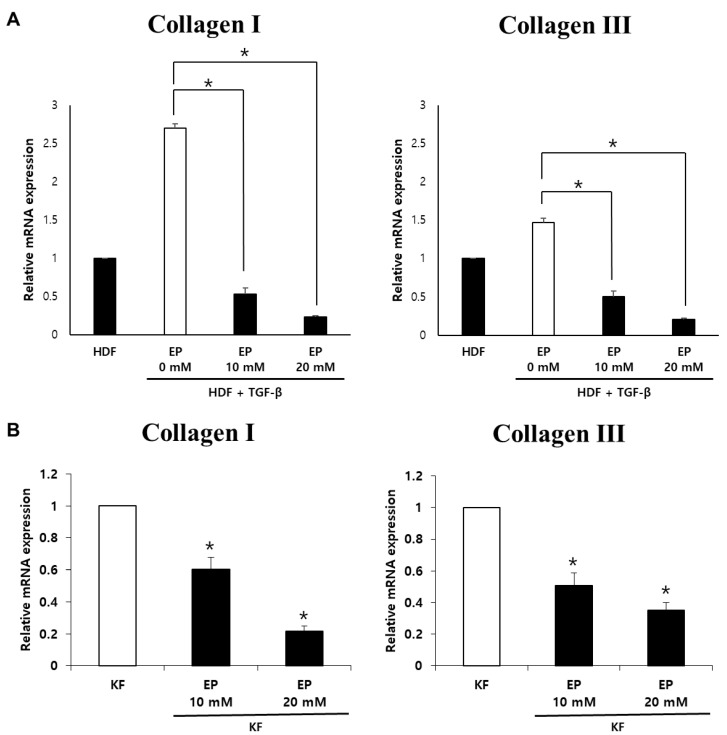
Effects of ethyl pyruvate on the expression of fibrotic and cytostatic markers in HDFs and KFs. (**A**) Treatment of TGF-β significantly increased the expression of collagen I/III mRNA. Treating ethyl pyruvate significantly decreased mRNA expression as the dose increased. (**B**) Similarly, treating ethyl pyruvate to KFs showed reduced collagen I/III mRNA expression (* *p* < 0.05).

We investigated the potential of EP to mitigate collagen synthesis and deposition in TGF-β1 (10 ng/mL)-treated HDFs and KFs. We measured the mRNA expression levels of collagen types I and III using quantitative polymerase chain reaction (qPCR). Following treatment with 20 mM EP, the expression of type I collagen mRNA in TGF-β1-treated HDFs and KFs was significantly reduced by 91% and 78%, respectively, compared to untreated cells (Figure 3). Similarly, type III collagen mRNA expression saw a significant decrease of 85% and 64%, respectively, in comparison to untreated HDFs and KFs (Figure 3). These results indicate that mRNA levels of collagen types I and III in TGF-β1-treated HDFs and KFs were significantly reduced upon treatment with EP [29].

### 2.4. Ethyl Pyruvate Suppressed the Collagen Type I, Collagen Type III, TGF-β1, and pSmad2/3 Complex in KFs

**Figure 4:**
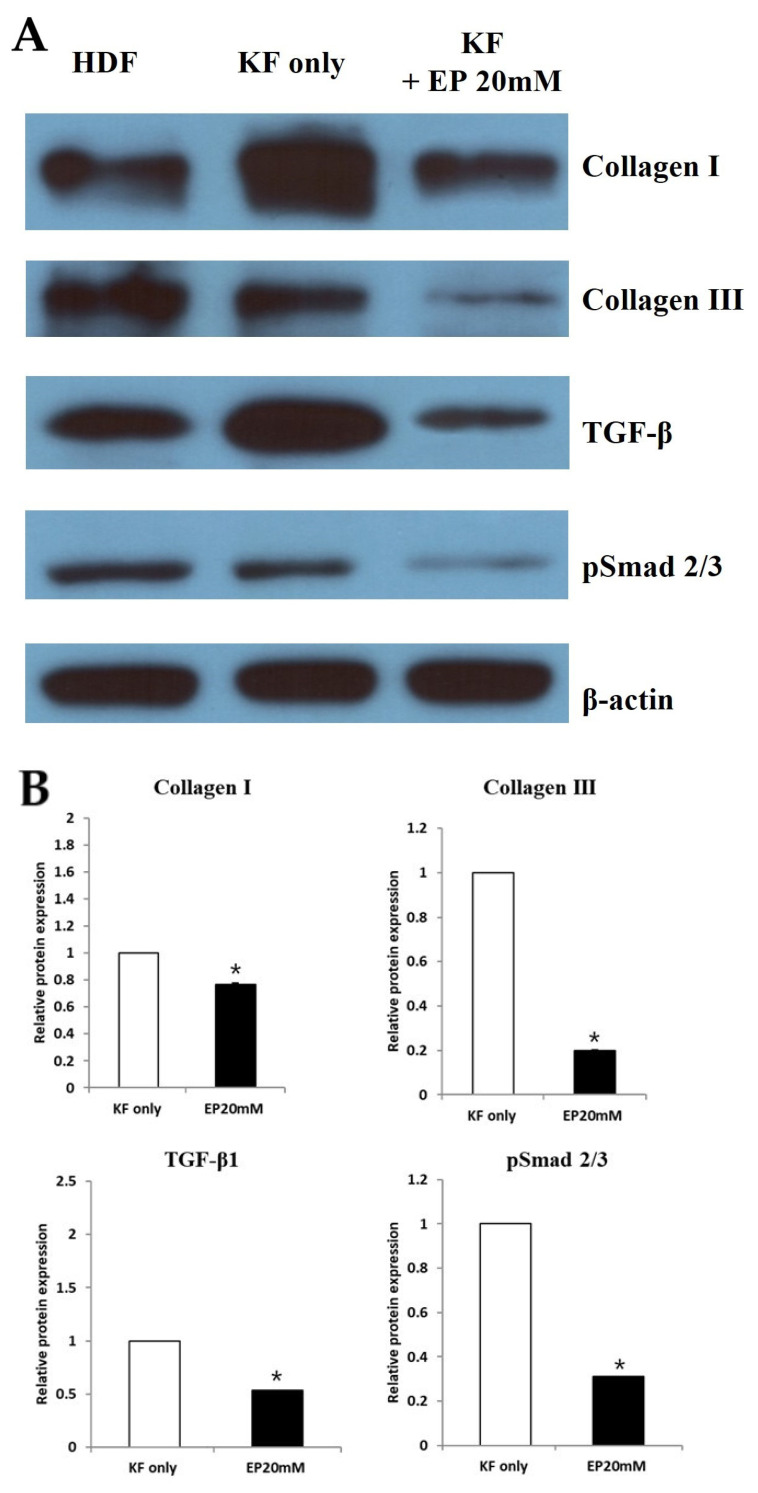
Effect of ethyl pyruvate on expression of fibrosis-related protein. (**A**) Western blot analysis of collagen I, III, TGF-β, and p-SMAD2/3 expression was performed; (**B**) upon densitometric analysis, collagen I, III, TGF-β, and p-SMAD2/3 protein expression levels decreased when KF cells were treated with 20mM of ethyl pyruvate (* *p* < 0.05).

To confirm the results above, protein expression levels of collagen type I, collagen type III, TGF-β1, and pSmad2/3 complex were examined by western blotting using KFs treated with EP (20 mM) for 48 h. Figure 4A illustrates that KFs inherently express significant amounts of collagen type I, collagen type III, TGF-β1, and the pSmad2/3 complex proteins. Notably, the expression levels of these proteins were substantially diminished following treatment with EP. Densitometric analysis further confirmed that EP treatment led to a notable reduction in the expression of collagen type I and III proteins in treated KFs by 58.1% and 76.9%, respectively (* *p* < 0.05; Figure 4B). Additionally, EP (20 mM) reduced TGF-β1 protein expression by 74% and that of the downstream pSmad 2/3 complex by 60% in treated KFs compared to untreated KFs (Figure 4B). These findings indicate that EP effectively inhibits the TGF-β1 signaling pathway in KFs.

### 2.5. Treatment of Ethyl Pyruvate Affects MMP-1 and MMP-3 mRNA Expression in HDFs and KFs

**Figure 5:**
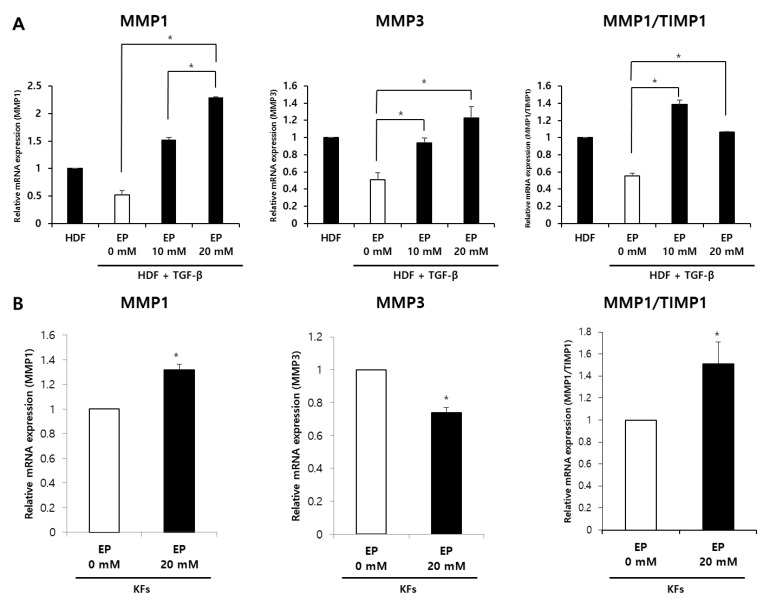
Effects of ethyl pyruvate on the mRNA expression of MMP1 and MMP3 mRNA levels in TGF-β1-treated HDFs and KFs. (**A**) MMP1 and MMP3 mRNA levels in TGF-β1-treated HDFs increased sequentially according to the concentration of ethyl pyruvate (0 mM, 10 mM, and 20 mM). (**B**) MMP1 mRNA levels in KFs increased by treatment of ethyl pyruvate. The ratio of MMP1/TIMP1 was significantly increased by EP (20 Mm) treatment in the TGF-β1-treated HDFs and KFs (* *p* < 0.05).

Metalloproteinases (MMPs) are enzymes secreted by dermal fibroblasts to regulate ECM remodeling, and our previous work has demonstrated that the activity of HMGB1 can influence their expression [16]. To further explore the impact of EP on the expression of ECM-degrading enzymes in keloids, we assessed the mRNA expression levels of metalloproteinase I (MMP1), metalloproteinase III (MMP3), and tissue inhibitor of metalloproteinases I (TIMP1) in both TGF-β1-treated HDFs and KFs. In TGF-β1-treated HDFs, MMP-1 mRNA levels were significantly upregulated by 4.4-fold following treatment with 20 mM EP compared to untreated HDFs. Similarly, MMP-3 mRNA levels were significantly increased by 2.4-fold in EP-treated HDFs (Figure 5A; * *p* < 0.05). The MMP1/TIMP1 ratio was notably elevated in TGF-β1-treated HDFs after 20 mM EP treatment. Moreover, in EP-treated KFs, mRNA expression of MMP1 was increased by 1.3-fold, whereas MMP3 mRNA expression was somewhat reduced by 20 mM EP treatment (Figure 5B). Nonetheless, the MMP1/TIMP1 ratio was significantly higher. These findings indicate that EP treatment leads to the upregulation of MMP-1 mRNA expression and MMP1/TIMP1, which play crucial roles in collagen degradation. 

### 2.6. Ethyl Pyruvate Suppresses Collagen I and III, Fibronectin, and Elastin in Keloid Spheroids

**Figure 6:**
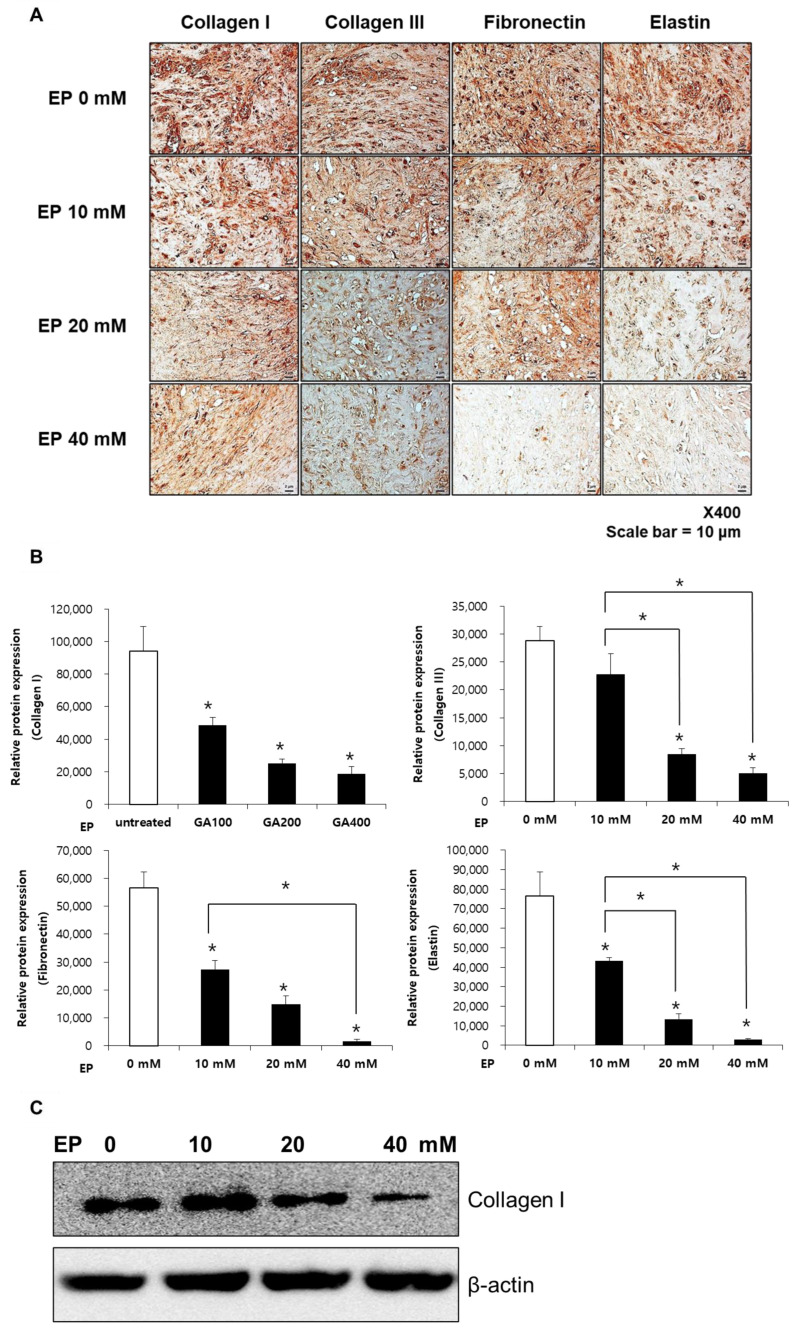
Immunohistochemical analysis of collagen I and III, fibronectin, and elastin in ethyl pyruvate-treated keloid spheroids (*n* = 3). (**A**) Representative images of collagen I and III, fibronectin, and elastin IHC staining of keloid spheroids treated with ethyl pyruvate (10, 20, and 40 mM). (**B**) Semi-quantification of protein expression is shown. Collagen I and III, fibronectin, and elastin were significantly decreased in keloid spheroids following ethyl pyruvate application. Asterisks (*) over each column signify a statistically significant difference compared to the control (0 mM, white bar) group (* *p* < 0.05). (**C**) Reduction in collagen I protein expression can be seen with western blot analysis.

Keloid spheroids from active-stage keloid patients (*n* = 3) were cultured and exposed to varying concentrations of EP to evaluate the impact on ECM expression. The expression levels of collagen types I and III, fibronectin, and elastin were analyzed using immunohistochemistry (Figure 6A). Quantitative analysis indicated that EP treatment led to a dose-dependent decrease in the expression of these ECM components. Specifically, treatment with 40 mM EP resulted in reductions of 80.3% for collagen I, 82.7% for collagen III, 97.1% for fibronectin, and 96.1% for elastin (Figure 6B). These findings collectively suggest that EP effectively modulates ECM production, contributing to the reduction in fibrosis in keloids. Furthermore, the decreased expression of collagen type I in EP-treated keloid spheroids from an active-stage keloid patient was verified through western blot analysis (Figure 6C), aligning with the results from the immunohistochemical analysis.

### 2.7. Ethyl Pyruvate Suppresses HMGB1, ERK1/2, and AKT Expression in Keloid Spheroids

**Figure 7:**
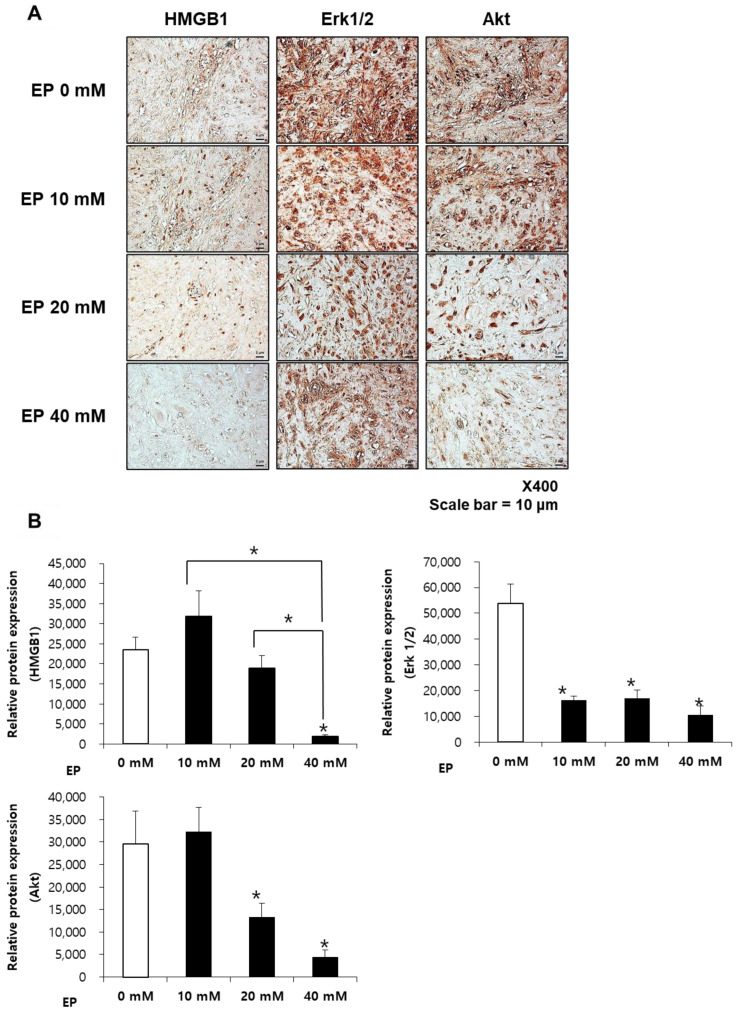
Effects of ethyl pyruvate on the expression of profibrotic factors in keloid spheroids. (**A**) Representative images of HMGB1, ERK 1/2, and AKT IHC staining of keloid spheroids treated with ethyl pyruvate (10, 20, and 40 mM). (**B**) Semi-quantification of protein expression is shown. HMGB1, ERK1/2, and AKT expression were significantly decreased after treatment with ethyl pyruvate. Asterisks (*) over each column signify a statistically significant difference compared to the control (0 mM, white bar) group. (* *p* < 0.05).

Intracellular signaling molecules, such as ERK1/2 and AKT, are involved in collagen synthesis and myofibroblast differentiation [16]. We explored the relationship between HMGB1 activation and these signaling molecules in keloid spheroids post-treatment with EP. Using immunohistochemistry, we assessed the expressions of HMGB1, ERK1/2, and AKT in the spheroids (Figure 7A). The results showed a significant reduction in HMGB1 expression following treatment with 20 mM and 40 mM EP. Similarly, the expression levels of the intracellular signaling molecules ERK1/2 and AKT, which are implicated in collagen synthesis and myofibroblast differentiation, were significantly decreased by treatments of 20 mM and 40 mM EP (* *p* < 0.05; Figure 7B). These findings, in conjunction with the previous results, indicate that EP treatment curtails ECM production by inhibiting profibrotic pathways.

## 3. Discussion

Excessive accumulation of ECM and hyperproliferation of fibroblasts are the primary pathophysiological causes of keloid formation. Multiple etiological factors often contribute to this process, involving the regulation of local inflammation, cellular proliferation, and apoptosis. Regulating these pathways could provide a promising target for keloid management.

Pyruvate is a potent and effective ameliorator of inflammatory injuries, exhibiting antioxidative, antitumor, and anti-inflammatory characteristics. However, its application has been largely limited due to its easy conversion into a potentially toxic metabolite [30]. EP, a simple derivative of pyruvate, is not inferior in terms of ameliorating inflammatory responses induced by sepsis, ischemia-reperfusion, radiation, and drug-induced injuries, and it is also safe and stable [31]. Previous studies have shown that EP treatment decreased fibrosis in the heart, liver, and lungs in animal models [12,13,32]. Thus, we hypothesized that EP treatment could reduce fibrosis by suppressing cellular proliferation and ECM formation.

EP reduces fibrosis via the downregulation of pro-fibrotic signaling. This has been previously reported in studies where ADR-induced cardiomyocytes were treated with EP. Fibrotic changes were alleviated by the upregulation of MMP expression and a higher MMP/TIMP ratio [12]. Additionally, suppression of TGF-β reduced fibrotic changes in human keratinocytes [33]. Treating bleomycin-induced bronchiolar cells with EP showed reduced levels of HMGB1, known to directly stimulate fibroblast proliferation, resulting in fewer fibrotic changes [14]. In our previous study, we demonstrated the overexpression of HMGB1 in keloid tissue, and that cytoplasmic translocation and exogenous HMGB1 could trigger proliferation of HDFs and enhance ECM production [16,23]. Although not statistically significant, EP treatment tended to reduce cytosolic HMGB1 fractionation. We were able to show that the exogenous secretion of HMGB1 was significantly decreased after EP treatment, which aligns well with previous reports [25]. 

Another possible explanation for the anti-fibrotic property of EP is its suppression of the TGF-β pathway. EP treatment of irradiated lung tissue showed decreased collagen synthesis through the suppression of TGF-β [32]. In this study, TGF-β-treated HDFs showed increased cellular viability similar to KFs, with an increase in factors involved in ECM production, such as collagen synthesis, while the MMP/TIMP ratio worsened due to the degradation of these factors. Treating both TGF-β-treated HDFs and KFs with EP decreased cellular viability, indicating that EP suppressed cellular proliferation in a dose-dependent manner. Furthermore, when examining the relative mRNA expression of collagen I/III, EP treatment showed lower levels at higher concentrations.

Proteolytic enzymes, such as MMPs, also play a role in keloid formation. In normal wound remodeling, MMPs degrade collagen and other ECM substrates, and their activity is regulated by TIMPs [34]. However, in keloid fibroblasts, MMP-1, which cleaves collagen types I and III, is decreased, while MMP-3, a stromelysin, is increased [35,36]. Overall, the balance of the MMP-to-TIMP ratio is thought to be important in scar formation, with a higher ratio resulting in reduced scarring [37]. In this study, we confirmed that EP treatment significantly increased the expression of MMP-1 and the MMP-to-TIMP ratio. However, for MMP-3, there was a difference between TGF-β-treated HDFs and KFs. EP upregulated MMP-3 expression in TGF-β-treated HDFs but reduced its expression in KFs. Since TGF-β signaling reduces MMP-3 expression, this could be attributed to the negative regulatory effect of EP on TGF-β signaling. In KFs, however, more complex factors could be involved [34].

In keloid spheroids, higher concentrations of EP were used to further validate the process. Immunohistochemistry of ECM elements showed decreased production of collagen I/III, fibronectin, and elastin. This suggests that EP treatment suppresses the overall formation of ECM. Additionally, pivotal mediators of pro-fibrotic signaling, Erk 1/2 and Akt, showed significant suppression after EP treatment. These results indicate that EP can exhibit anti-fibrotic effects on keloids. 

Previous research has shown that through various pharmacological and biological activities, EP decreases inflammation, oxidative stress, and oncogenesis [38,39]. The suppression of fibrosis and over-proliferation in keloids are not the only complementary factors contributing to the anti-fibrotic action of EP. Therefore, we aim to further advance research to elucidate the effects of EP on keloid tissues and the mechanism of keloid formation. 

The limitation of this study is that it is difficult to confirm the exact molecular pathway with the results depicted in this research. Studies have shown HMGB1’s abundance in keloid tissues and its role in inducing fibrosis through TGF-β1, Erk 1/2, Akt, and NF-κB pathways [35,36,40,41]. Although Erk1/2 and Akt were decreased with EP treatment, this does not fully reflect the actual level of the signaling activity. Future studies should include staining for the active phosphorylated form of these proteins, along with other apoptotic markers and signaling pathways, to determine whether EP effectively reduces keloids.

To our knowledge, this is the first study to demonstrate the anti-fibrotic effects of EP on keloid fibroblasts, suggesting potential therapeutic benefits in keloid treatment by regulating cell death and pro-fibrotic activities.

## 4. Materials and Methods

### 4.1. Preparation of Cells 

HDFs and KFs were obtained from the American Type Culture Collection (Manassas, VA, USA). Cells were cultured in Dulbecco’s Modified Eagle’s Medium (Gibco, Grand Island, NY, USA) containing 10% fetal bovine serum (Sigma-Aldrich, St. Louis, MO, USA), penicillin (30 U/mL), and streptomycin (300 µg/mL). Cells were maintained at 37 °C in a humidified incubator under 5% CO_2_, and the medium was changed every 2 days. For all experiments, cells were used before passage #7. To induce fibroblasts, HDFs were treated with TGF-β at a concentration of 10 ng/mL and cultured for 48 h. 

### 4.2. Preparation of Keloid Spheroids

Keloid and adjacent normal dermal tissues were obtained during surgical procedures from three patients with active-stage keloids (Table 1). Human keloid tissue samples were collected after obtaining written informed consent and in accordance with a protocol approved by the Yonsei University College of Medicine Institutional Review Board (IRB No. 4-2015-0228). Keloid spheroids were prepared by dissecting the central dermal tissue of the keloids into 2 mm diameter pieces using sterile 21-gauge needles. The explants were plated onto HydroCell^®^ 24 multi-well plates (Nunc, Rochester, NY, USA) and cultured for 4 h in Iscove’s Modified Dulbecco’s Medium (Gibco) supplemented with 5% fetal bovine serum, 10 μM insulin, and 1 μM hydrocortisone. Ethyl pyruvate, an inhibitor of HMGB1, was added to the plates containing keloid spheroids at concentrations of 0, 1, 10, 20, and 40 mM, and incubated at 37 °C in 5% CO_2_ for 48 h. The treated keloid spheroids were then fixed with 4% formalin, paraffin-embedded, and sectioned into 5 μm thick slices.

### 4.3. Methyl Thiazolyl-Diphenyl-Tetrazolium Bromide (MTT) Assay 

To assess cellular viability and cellular proliferation after ethyl pyruvate treatment of KFs and HDFs, a 3-(4,5-dimethylthiazol-2-yl)-2,5-diphenyltetrazolium bromide (MTT) assay was performed. In this assay, 1 × 10^4^ cells/cm^2^ of KFs and HDFs were seeded in triplicate in 96-well plates. After exposing the cells to concentrations of 0, 1 mM, 5 mM, 10 mM, and 20 mM of ethyl pyruvate (Sigma-Aldrich) for 48 h, 200 μL of a 0.5 mg/mL MTT solution (Boehringer, Mannheim, Germany) was added to each well. The plates were then incubated at 37 °C for 3 h. To dissolve the resulting formazan, 200 μL of dimethyl sulfoxide (Sigma-Aldrich) was added to each well after removing the MTT solution. The absorbance was measured at 570 nm using a microplate reader (Bio-Rad, Hercules, CA, USA).

### 4.4. Western Blotting Analysis for Collagen Markers and Profibrotic Markers

Quantitative measurement of representative profibrotic markers was performed using western blotting. HDFs and KFs treated with 0 mM, 10 mM, 20 mM, and 40 mM ethyl pyruvate (cells were seeded at 1 × 10^5^ cells/well) were cultured in 100 mm × 20 mm dishes for 48 h. The cells were cultured in triplicate. Cells were lysed in 50 mM Tris-HCl (pH 7.6), 1% Nonidet P-40, 150 mM NaCl, and 0.1 mM zinc acetate in the presence of protease inhibitors. Protein concentrations were measured by the Lowry method (Bio-Rad), and 20 µg of each sample was loaded into a 10% sodium dodecyl sulfate–polyacrylamide gel for electrophoresis. Proteins were transferred onto a polyvinylidene difluoride membrane (Millipore, Billerica, MA, USA) by electrophoresis. The membrane was blocked with blocking buffer for 1 h and incubated with mouse anti-HMGB1 (Abcam, Cambridge, UK), mouse anti-histone (Abcam), and rabbit anti-α-tubulin (Santa Cruz Biotechnology) overnight at 4 °C. After 2 h of incubation at room temperature with the secondary antibody (horseradish peroxidase-conjugated anti-rabbit or anti-mouse; Santa Cruz Biotechnology, Dallas, TX, USA), protein bands were visualized using chemiluminescence reagents (Amersham Pharmacia Biotech, Piscataway, NJ, USA) according to the manufacturer’s instructions. Protein expression was analyzed using ImageJ 1.51f software (National Institutes of Health, Bethesda, MD, USA).

### 4.5. Enzyme-Linked Immunosorbent Assay (ELISA)

HDFs (2 × 10^5^ cells) were seeded in 6 cm culture dishes. LPS-treated HDFs were treated with 20 mM of ethyl pyruvate. Forty-eight hours after treatment, supernatants were collected and centrifuged at 15,000× *g* for 10 min at 4 °C. The secreted HMGB1 protein in the supernatants was measured using an ELISA kit (R&D Systems, Minneapolis, MN, USA).

### 4.6. Quantitative Real-Time Polymerase Chain Reaction (qPCR)

Total RNA was extracted with TRIzol Reagent (Gibco, BRL), and complementary DNA was synthesized from 500 ng of RNA using the First-strand cDNA Synthesis kit (Promega Corp., Madison, WI, USA) as described in the manufacturer’s protocol. TaqMan primer/probe kits [assay ID: Hs00164004_m1 (collagen type I), Hs00164103_m1 (collagen type III), Hs00233958_m1 (MMP1), and Hs00233962_m1 (MMP3), Applied Biosystems, Foster City, CA, USA] were used for qPCR and measurements were taken using the ABI Prism 7500 HT Sequence Detection System (Applied Biosystems, Foster City, CA, USA). The expression of mRNA was normalized with glyceraldehyde-3-phosphate dehydrogenase (GAPDH) as the endogenous control (assay ID: Hs99999905_m1, Applied Biosystems, Foster City, CA, USA).

### 4.7. Histology and Immunohistochemistry (IHC) of Keloid Spheroids 

Keloid spheroids were treated with 0, 10, 20, and 40 mM of ethyl pyruvate for 48 h. The spheroids were then washed, fixed with 4% formalin, paraffin-embedded, and sectioned into 5 μm thick slices. For IHC staining, the keloid spheroid sections were incubated overnight at 4 °C with primary antibodies including mouse anti-HMGB1 (Abcam), mouse anti-AKT (Abcam), mouse anti-ERK1/2 (Abcam), mouse anti-collagen type I (Abcam), mouse anti-collagen type III (Sigma-Aldrich), mouse anti-elastin (Sigma-Aldrich), and mouse anti-fibronectin (Santa Cruz Biotechnology). The sections were then incubated at room temperature for 20 min with the Envision™ kit (Dako, Glostrup, Denmark) following the manufacturer’s instructions. Diaminobenzidine/hydrogen peroxidase (Dako) was used as the chromogenic substrate. All slides were counterstained with Mayer’s hematoxylin. The expression of the related proteins was semi-quantitatively analyzed using Metamorph^®^ image analysis software version 7.1. Results are expressed as the mean optical density of different digital images.

### 4.8. Statistical Analysis

Results are expressed as means ± standard error of the mean (SEM). Data were analyzed by a repeated-measures one-way ANOVA. A paired *t*-test was used to analyze statistical differences between the two groups. Results were judged significant when *p* < 0.05. 

## 5. Conclusions

To conclude, the current study demonstrated that EP exerts anti-fibrotic effects by downregulating pro-fibrotic signaling pathways, including TGF-β. EP treatment also increased MMP1 expression, improved the MMP-to-TIMP ratio, and suppressed ECM formation, suggesting potential therapeutic benefits in keloid treatment.

## Figures and Tables

**Table 1:** Demographic information and description of keloids obtained from subjects.

	Sex	Race	Age (Years)	Location	Types
1	F	Korean	48	Earlobe	Pedunculated
2	M	Korean	22	Earlobe	Pedunculated
3	F	Korean	35	Shoulder	Sessile

## Data Availability

The data that support the findings of this study are available from the corresponding author upon reasonable request.
